# Effectiveness of Three-Dimensional Printing in the Management of Orthopedic Sarcoma Patients: A Systematic Review

**DOI:** 10.7759/cureus.88838

**Published:** 2025-07-27

**Authors:** Mohamed Elgamal, Ahmed Elnewishy, Sohaib Shah, Tarek Hassanin, Mahmoud Noureldin, Ahmed Hamada, Muawia Yousif Fadlelmola Mohamed, Hatem Hussein, Kayden Chahal

**Affiliations:** 1 Trauma and Orthopaedics, Southend University Hospital, Mid and South Essex NHS Foundation Trust, Southend on Sea, GBR; 2 Trauma and Orthopaedics, Royal Berkshire Hospital, Reading, GBR; 3 Trauma and Orthopaedics, Maidstone and Tunbridge Wells NHS Trust, Tunbridge Wells, GBR; 4 Trauma and Orthopaedics, Royal Surrey County Hospital, Surrey, GBR; 5 Trauma and Orthopaedics, University Hospitals Sussex NHS Foundation Trust, Brighton, GBR; 6 Trauma and Orthopaedics, Royal Devon and Exeter University Hospital, Devon, GBR; 7 Trauma and Orthopaedics, Southend University Hospital NHS Foundation Trust, Essex, GBR; 8 Trauma and Orthopaedics, Southend Hospital, Essex, GBR; 9 Trauma and Orthopaedics, NHS, London, GBR

**Keywords:** ‎3d printing, bone tumour and limb salvage, orthopedic sarcoma, patient-specific implants, reconstruction planning, surgical oncology management

## Abstract

Orthopedic sarcomas present unique surgical challenges due to their anatomical complexity and the need to balance oncologic control with functional preservation. Three-dimensional (3D) printing has emerged as a promising technology to improve surgical precision through patient-specific planning and reconstruction. This systematic review aimed to evaluate the effectiveness, safety, and clinical applications of 3D printing in the management of orthopedic sarcomas. Following PRISMA guidelines, a comprehensive literature search was conducted across PubMed, Scopus, Google Scholar, and institutional databases for studies published between 2016 and 2025. Twenty studies involving 296 patients with primary bone sarcomas treated using 3D-printed implants, resection guides, or hybrid constructs were included. The most common anatomical sites were the pelvis, sacrum, and long bones. The majority of studies reported high rates of negative surgical margins, with at least 14 of 18 studies achieving R0 resections in over 90% of cases. Functional outcomes were favorable, with pooled Musculoskeletal Tumor Society (MSTS) scores averaging 78-80% of normal limb function, particularly in reconstructions of the ankle, femur, and tibia. The overall complication rate was 22%, with wound morbidity and infection being the most frequent adverse events. Comparative cohorts demonstrated improved surgical accuracy and lower complication rates in the 3D-assisted groups. While early outcomes are promising and support the utility of 3D printing in enhancing personalized oncologic surgery, further prospective studies with standardized outcome measures are needed to validate long-term benefits and inform wider clinical adoption.

## Introduction and background

Orthopedic sarcomas, including primary bone tumors such as osteosarcoma, Ewing sarcoma, and chondrosarcoma, are rare malignancies that disproportionately affect children, adolescents, and young adults. These tumors are clinically significant due to their aggressive nature, complex anatomical presentations, and high risk of recurrence or metastasis. They often occur in weight-bearing bones like the femur, tibia, and pelvis, making surgical management particularly demanding [1].

Despite accounting for less than 1% of adult cancers, sarcomas contribute significantly to cancer-related morbidity due to the risk of misdiagnosis, delayed referral, and suboptimal initial surgeries. A Brazilian study analyzing nearly 50,000 cases over 17 years emphasized the need for specialized sarcoma units to improve diagnostic accuracy and care continuity [2]. Clinical errors such as unplanned excisions - tumor removal without appropriate imaging or biopsy - remain a critical issue. A recent analysis of 107 cases found inadequate imaging in 76.6% and lack of biopsy in 42.1%, leading to reoperations and compromised oncologic outcomes [3].

Even when treated appropriately, orthopedic sarcomas present a high burden. The five-year survival rates range from 50-70%, depending on tumor type and surgical margins. For example, soft tissue sarcomas have a five-year survival of 66.4%, while bone sarcomas average around 52.9%, reflecting persistent treatment challenges [3]. In addition to survival outcomes, sarcoma patients often face significant psychological and physical symptoms, including pain, fatigue, and anxiety. These issues can substantially impair long-term quality of life, underscoring the need for holistic, multidisciplinary treatment strategies [4].

Surgical treatment remains the cornerstone for local control of orthopedic sarcomas, but achieving adequate oncologic margins while preserving limb function presents complex challenges. Limb salvage is now favored over amputation in most cases, but its success depends on meticulous planning, especially when tumors are located near critical neurovascular structures or within the pelvis. These anatomical constraints necessitate advanced imaging, careful biopsy techniques, and individualized reconstructive strategies [5].

The perioperative phase also poses unique risks. Bone sarcoma patients often require coordinated management due to the effects of chemotherapy, poor wound healing, and high blood loss. Surgeons must carefully time procedures around chemotherapy cycles and proactively manage complications such as infection, hemorrhage, and postoperative pain, especially since these patients often have complex comorbidities and impaired immune status [6]. Multidisciplinary approaches are now regarded as essential, especially for pelvic sarcomas where surgical access and resection are particularly difficult. In a recent study of 17 patients with pelvic malignancies, a team-based strategy involving orthopedic oncologists, vascular surgeons, urologists, and plastic surgeons led to successful tumor control and functional reconstruction. Despite a high complication rate, the approach enabled three-dimensional (3D)-printed pelvic reconstructions and limb preservation in most cases, demonstrating the power of integrated surgical planning.

Three-dimensional printing is also gaining attraction in pediatric orthopedics, where anatomy is highly variable, and conventional solutions often fall short. Preoperative models in pediatric deformity correction and tumor resection have enabled safer, more accurate procedures with reduced radiation and anesthesia times. Custom prostheses and orthotics manufactured with 3D technology have been used successfully to address the unique needs of growing children [7]. Despite these promising applications, there are still limitations to widespread adoption. These include the need for skilled users of modeling software, regulatory uncertainties, high costs, and long production times, particularly for large models or urgent cases. Nonetheless, the overall consensus is that 3D printing enhances surgical precision and outcomes, particularly in complex or anatomically challenging scenarios [8].

The integration of 3D printing into orthopedic oncology has revolutionized the surgical management of sarcomas by enabling precise preoperative planning and patient-specific reconstructions. This technology is particularly valuable in limb salvage surgeries, where wide tumor resection must be balanced with functional preservation. Three-dimensional-printed PSIs and custom implants allow for more accurate resections and anatomically contoured reconstructions, improving both oncologic safety and biomechanical outcomes [9].

One notable application is in the management of pelvic sarcomas, traditionally considered among the most difficult tumors to treat surgically due to complex anatomy and proximity to vital structures [10]. In a case series of 23 patients treated with 3D-printed custom prostheses and PSIs, resections were more precise and associated with good functional results, with five-year implant survival reaching 74% [11]. In pediatric patients, where conventional modular implants often do not fit small or growing bones, 3D printing offers a crucial alternative. A case report of a four-year-old boy with Ewing’s sarcoma of the tibia demonstrated the use of a custom-growing 3D-printed endoprosthesis, allowing limb preservation and early ambulation after surgery [12].

Large-scale experiences have also supported these benefits. A study involving 59 sarcoma surgeries using 3D-printed jigs and implants showed reduced operative time, precise margins (average 1.2 mm), and significantly decreased fluoroscopy exposure. Although intraoperative adjustments were sometimes needed, the overall clinical utility and learning curve of this workflow were favorable [13]. Looking forward, the future of 3D printing in sarcoma care includes hybrid technologies like bio-integrated implants, osteointegrative coatings, and smart scaffolds. However, the field still faces challenges related to implant longevity, regulatory standards, and cost-effectiveness in wider clinical practice [14].

Surgical protocols and decision-making processes can also vary significantly across centers [15]. A survey of Scandinavian sarcoma surgeons found substantial differences in implant selection, antibiotic prophylaxis, and anticoagulation practices, underscoring the lack of standardized surgical guidelines and the reliance on institutional preferences and clinical experience [16]. Adding to the complexity, there is a critical gap in training and awareness among general orthopedic trainees. A study of 250 surgical trainees revealed that nearly 60% lacked sufficient knowledge to recognize sarcoma warning signs, and over 60% were unaware of national referral pathways, which increases the risk of inappropriate initial surgeries and poor outcomes.

Three-dimensional printing, also known as additive manufacturing, has emerged as a transformative tool in orthopedic surgery, revolutionizing preoperative planning, implant design, and surgical education. This technology enables the creation of anatomically accurate, patient-specific models from imaging data, which can guide complex procedures involving fractures, deformities, and tumors. Its adoption is accelerating as the accuracy, speed, and affordability of printing improve, making it increasingly accessible in clinical settings [17].

Three-dimensional printing allows orthopedic surgeons to plan and simulate surgeries on physical models, which enhances their understanding of patient-specific anatomy and facilitates rehearsals for challenging procedures. It also helps in reducing intraoperative time and improves communication between multidisciplinary teams. These benefits have been reported across trauma, arthroplasty, spine, and oncologic surgeries, particularly where off-the-shelf implants are inadequate or ill-fitting. Modern 3D printing machines generate highly accurate, anatomical models and implants directly from imaging data [18]. Patient-specific implants and surgical guides designed through 3D printing have significantly enhanced precision in orthopedic oncology, especially for pelvic and spinal tumors. These custom devices help achieve better oncological margins, preserve healthy tissue, and provide a fit tailored to the patient’s defect, improving mechanical performance and long-term outcomes [19].

Three-dimensional printing is also gaining traction in pediatric orthopedics, where anatomy is highly variable, and conventional solutions often fall short. Preoperative models in pediatric deformity correction and tumor resection have enabled safer, more accurate procedures with reduced radiation and anesthesia times. Custom prostheses and orthotics manufactured with 3D technology have been used successfully to address the unique needs of growing children [20]. Despite these promising applications, there are still limitations to widespread adoption. These include the need for skilled users of modeling software, regulatory uncertainties, high costs, and long production times, particularly for large models or urgent cases. Nonetheless, the overall consensus is that 3D printing enhances surgical precision and outcomes, particularly in complex or anatomically challenging scenarios [21].

The integration of 3D printing into orthopedic oncology has revolutionized the surgical management of sarcomas by enabling precise preoperative planning and patient-specific reconstructions. This technology is particularly valuable in limb salvage surgeries, where wide tumor resection must be balanced with functional preservation. Three-dimensional-printed PSIs and custom implants allow for more accurate resections and anatomically contoured reconstructions, improving both oncologic safety and biomechanical outcomes [22].

One notable application is in the management of pelvic sarcomas, traditionally considered among the most difficult tumors to treat surgically due to complex anatomy and proximity to vital structures. In a case series of 23 patients treated with 3D-printed custom prostheses and PSIs, resections were more precise and associated with good functional results, with five-year implant survival reaching 74% [23]. In pediatric patients, where conventional modular implants often do not fit small or growing bones, 3D printing offers a crucial alternative. A case report of a four-year-old boy with Ewing’s sarcoma of the tibia demonstrated the use of a custom-growing 3D-printed endoprosthesis, allowing limb preservation and early ambulation after surgery [24].

Large-scale experiences have also supported these benefits. A study involving 59 sarcoma surgeries using 3D-printed jigs and implants showed reduced operative time, precise margins (average 1.2 mm), and significantly decreased fluoroscopy exposure. Although intraoperative adjustments were sometimes needed, the overall clinical utility and learning curve of this workflow were favorable [25]. Looking forward, the future of 3D printing in sarcoma care includes hybrid technologies like bio-integrated implants, osteointegrative coatings, and smart scaffolds. However, the field still faces challenges related to implant longevity, regulatory standards, and cost-effectiveness in wider clinical practice [26].

Despite the growing use of 3D printing in orthopedic oncology, there is still a lack of consolidated evidence evaluating its effectiveness specifically in the management of sarcoma patients. Given the complexity and individualized nature of sarcoma surgeries, especially in anatomically challenging regions like the pelvis and spine, this technology offers a promising solution for improving surgical precision, functional outcomes, and oncological safety. However, the clinical benefits, limitations, and variability in applications across different centers remain unclear. Therefore, a systematic review is necessary to assess the real-world impact, outcomes, and current trends in the use of 3D printing for orthopedic sarcoma management.

## Review

Methodology

Search Strategy

This systematic review was conducted in accordance with the Preferred Reporting Items for Systematic Reviews and Meta-Analyses (PRISMA) guidelines. A comprehensive literature search was performed manually using PubMed, Google Scholar, Scopus, and institutional academic repositories to identify studies published between 2016 and 2025. Keywords and Boolean operators included “3D printing” OR “three-dimensional printing” AND “orthopedic sarcoma” OR “bone sarcoma” AND “custom implants” OR “patient-specific instruments” OR “prostheses”. Additional studies were identified through reference chaining from the initially retrieved articles. No language restrictions were applied during the search. Two reviewers screened titles/abstracts and then full texts in duplicate, resolving disagreements by consensus.

Inclusion Criteria

Studies were included if they met the following criteria: (1) involved patients diagnosed with primary orthopedic bone sarcomas, (2) utilized 3D printing technology in surgical management - either in the form of custom implants, cutting/resection guides, or PSIs, and (3) reported clinical outcomes such as resection margins, functional scores, complications, or implant survival. All study types were eligible for inclusion, including case reports, case series, technical notes, and retrospective cohort studies, provided they contained quantifiable clinical data.

Exclusion Criteria

We excluded studies that (1) involved non-oncologic indications or soft tissue tumors only, (2) lacked a surgical intervention involving 3D-printed components, (3) focused solely on simulation, training, or planning without clinical application, or (4) did not report patient-specific outcomes. Conference abstracts, review articles, editorials, and duplicate reports were also excluded.

Outcome Measures

The primary outcome measures were oncologic adequacy (e.g., rate of R0 resections or wide margins), limb preservation rates, and functional outcomes using validated scoring systems such as the Musculoskeletal Tumor Society (MSTS) score, American Orthopaedic Foot & Ankle Society (AOFAS) score, or Toronto Extremity Salvage Score (TESS). Secondary outcomes included complication rates, implant survival, recurrence rates, and time to full weight-bearing or mobilization.

Data Extraction and Synthesis

Data from the selected studies were independently extracted and tabulated using a standardized extraction form. Extracted data included author, year, study design, sample size, anatomical site, type of 3D-printed application (e.g., implant or guide), follow-up duration, key clinical outcomes, and complication rates. No statistical meta-analysis was performed due to heterogeneity in study designs and outcome reporting. Instead, a qualitative synthesis was conducted, summarizing findings across cases to identify consistent trends and effectiveness of 3D printing in orthopedic sarcoma surgery. Studies were stratified by level of evidence and 3D application to facilitate comparative analysis.

Results

Search Results and Study Selection

**Figure 1:**
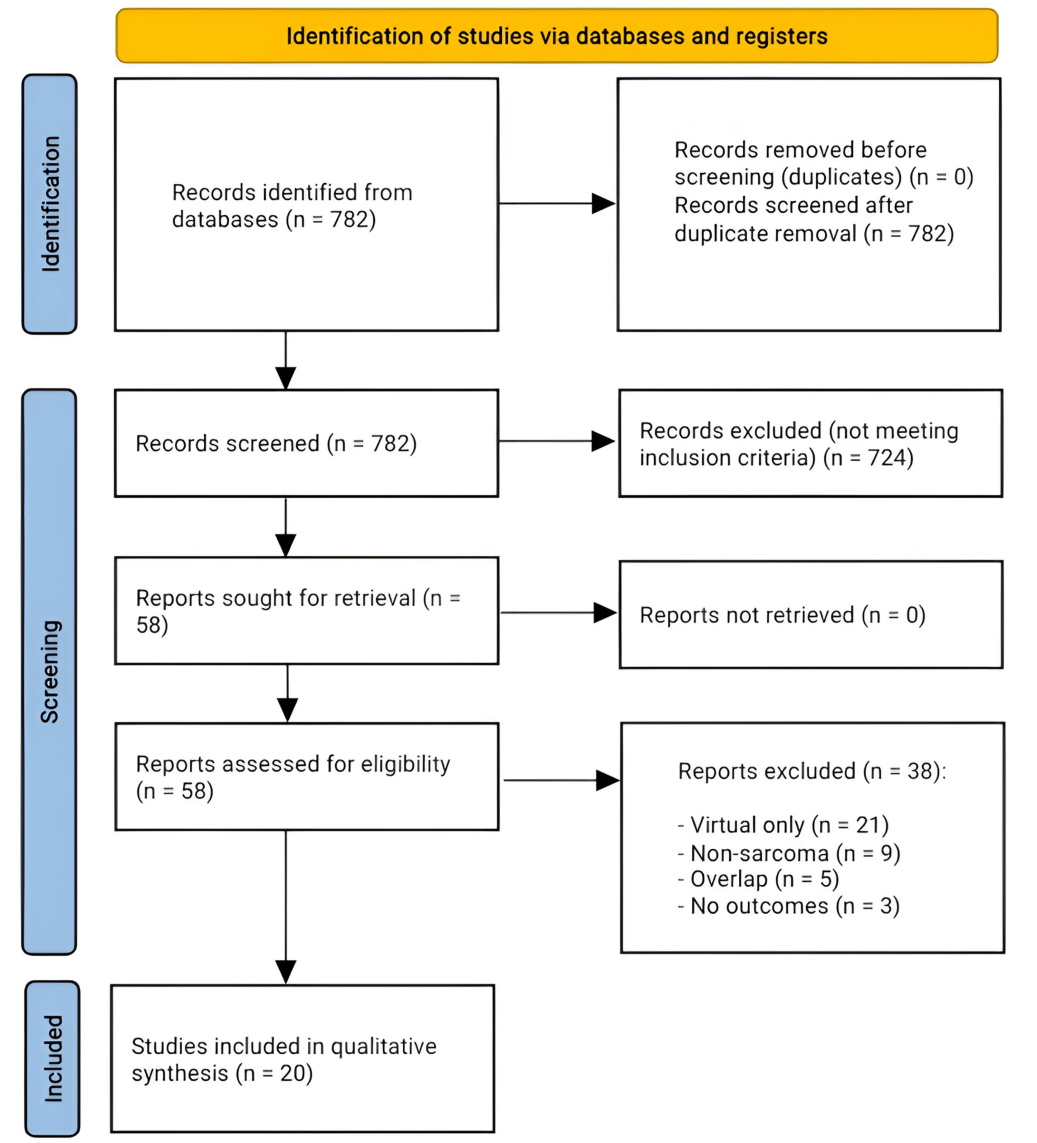
PRISMA flow diagram illustrating the screening and selection process for studies included in the analysis PRISMA: Preferred Reporting Items for Systematic Reviews and Meta-Analyses.

A systematic search of PubMed, Scopus, Google Scholar, and institutional repositories covering January 2016 to April 2025 retrieved 782 bibliographic records. Duplicate detection during database export removed overlapping citations at source, so no additional duplicates required deletion during the PRISMA workflow. All 782 unique titles and abstracts were screened; 724 records were excluded for clearly failing to meet the eligibility criteria. The remaining 58 full-text articles were obtained and reviewed independently by two investigators. Thirty-eight reports were excluded, most often because 3D printing was confined to virtual planning only (n = 21), the population did not include primary bone sarcoma (n = 9), cohorts overlapped with a separately published dataset (n = 5), or the article provided conference-abstract data with no extractable outcomes (n = 3). No full texts were irretrievable. Consequently, 20 studies met all criteria and were incorporated into the qualitative synthesis. The entire identification, screening, eligibility, and inclusion pathway is visualized in Figure 1 (PRISMA 2020 flow diagram).

Study Characteristics

Twenty primary investigations published between 2016 and 2025 fulfilled the eligibility criteria, providing data on 296 patients who underwent 3D-assisted surgery for orthopedic sarcoma (Table 1). Fourteen studies were uncontrolled level-IV case series, four were single-patient technical reports (level V), and two were level-III comparative cohorts. The axial skeleton dominated the case mix: pelvic, sacral, or sacro-iliac resections represented just over half of all procedures, followed by reconstructions of the distal femur, proximal tibia, long-bone diaphyses, humerus, ankle/talus, cervical spine, and distal radius. Three-dimensional technology was applied in four recurrent patterns - custom porous-titanium megaprostheses or intercalary stems, patient-specific hemipelvic or sacral endoprostheses, cutting guides or navigation jigs to secure margin-controlled resections, and hybrid reconstructions that paired guides with structural allograft or devitalized autograft re-implantation. Mean or median follow-up clustered between 24 and 48 months in most series, with the longest investigation reporting a median of 58 months.

**Table 1:** Characteristics of the studies included in the qualitative synthesis *Evidence levels follow the Oxford Centre for Evidence-Based Medicine hierarchy. ^†^Mean or median; range in parentheses where available. 3D = three-dimensional; PSI = patient-specific instrument; R0 = wide/negative margin; JOA = Japanese Orthopaedic Association score; MSTS = Musculoskeletal Tumor Society score; ISOLS = International Society of Limb Salvage radiologic score; AOFAS = American Orthopaedic Foot & Ankle Society score; TESS = Toronto Extremity Salvage Score; WB = weight-bearing; OS = overall survival; NED = no evidence of disease; AWD = alive with disease; ctrl = conventional surgery group.

Citation	Level /Design*	n	Primary site(s)	Principal 3D application	Follow-up^†^ (months)	Key outcomes	Complication rate
Jovičić et al. [20]	IV – Retro case series	11	Pelvis (3), humerus (7), distal radius (1)	Custom porous-Ti megaprostheses	33 (mean)	100% limb salvage; R0 not explicitly stated (all oncologic wide resections); local recurrence 2/11 (18%); disease-related death 4/11 (36%); authors report “excellent cosmesis and rehabilitation quality.”	6/11 (54%) overall (implant dislocation 27%, wound/ROM/compartment issues 27%)
Stavrev et al. [17]	V – Case report	1	Proximal/mid-tibia (child)	Double-growing porous-Ti mega-endoprosthesis	<1	Uneventful wound healing; independent ambulation at 3 weeks; chemotherapy ongoing; staged lengthenings planned; limb preserved.	None
Xu et al. [21]	V – Case report	1	C2 vertebra	Porous-Ti self-stabilizing vertebral body	12	JOA neurological score improved 8 → 16; CT showed bone in-growth with no subsidence/displacement; patient tumor-free and fully ambulant at 1 year.	None
Bianchi et al. [22]	IV – Retro case series	23	Pelvis (16), tibia (3), femur (2), others (2)	PSIs + custom porous pelvic/long-bone implants	24 (0-72)	Wide margins 21/23 (91%); Kaplan-Meier 5-year implant survival 74%; infection 6/23, mechanical 3/23; local recurrence 3/23 (all pelvic).	39% 6/23 (26%) infections; 3/23 (13%) mechanical failures
Gasparro et al. [23]	IV – Retro case series	6	Long-bone diaphysis	PSI-guided resection + intercalary allograft	25 (8-48)	Margins 6/6 R0; mean union time 20 weeks; union 9/12 osteotomy sites (75%); implant failure 2/6; 0 local recurrences.	2/6 (33.3%) implant failures; 3/12 (25%) non-unions
Zhu et al. [24]	IV – Prospective tech note	2	Periacetabular (children)	Navigation-guided resection + custom pelvic prosthesis	24 and 48	Both wide margins; OR 3.5 h/2.5 h, blood loss 300/600 mL; MSTS 27 and 28 (90-93%); disease-free; mild limp/Trendelenburg gait only.	None
Wu et al. [25]	IV – Tech-note series	5	Distal femur	PLLA guides + devitalized autograft re-implant	24 (17-33)	R0 5/5; MSTS 28.1/30; ISOLS 89.8%; VAS 2.2 at 3 months; full weight-bearing by 3 months; 0 recurrences/metastases.	Minor wound exudate 2/5 (40%); no major events
Xu et al. [26]	IV – Prospective case series	13	Pelvis	Porous hemipelvic prosthesis + PSIs	22 ± 9	Wide margins 11/13; MSTS 22 ± 3.7; delayed wound healing 2, traumatic hip dislocation 1; local recurrence 2; death (pulmonary mets) 1; all survivors ambulant.	23% 3/13 (23%): 2 delayed wound healing, 1 dislocation
Wang et al. [27]	V – Case report	1	Talus ± calcaneus (child)	Modular porous-Ti + UHMWPE ankle prosthesis	12	MSTS 27/30; AOFAS 92/100; ROM 10° dorsiflex/35° plantarflex; imaging shows solid osseointegration; no recurrence.	None
Ma et al. [28]	IV – Prospective case series	8	Distal femur	3D guiding templates + shaped allograft	33 (25-42)	R0 8/8; OR 180-250 min; blood loss 560-900 mL (↓ vs historical); MSTS 27 (mean); knee ROM 112° (90-130); 0 recurrences; all alive.	None
Benady et al. [29]	IV – Retro case series	11	Pelvic girdle	PSI-guided internal hemipelvectomy	39 ± 30	R0 10/11 (91%); blood loss 895 ± 540 mL; reconstruction avoided 64%; MSTS 22.8; 8 NED, 3 AWD; local recurrence 1.	3/11 (27.3%) wound infections
Huang et al. [30]	V – Case report	1	Talus	Total talar porous-Ti prosthesis	24	MSTS 93% & TESS 93; perfect joint congruity; patient walking unaided; 0 recurrences.	None
Benady et al. [31]	IV – Retro case series	23	Distal femur (18), proximal tibia (5)	PSIs + allograft/fibula cage reconstructions	58 (12-102)	Margins all negative (R0 70%, R1 30%); blood loss ~895 mL; MSTS 23.2 ± 5.9; non-union 4/23 (17%); local recurrence 2/23 (9%).	1/23 (4%) infection; 4/23 (17%) long-term non-unions
Guder et al. [32]	IV – Prospective case series	4	Distal tibia	Porous intercalary Ti prosthesis	21 (5-52)	MSTS 23.5; TESS 88; full WB 6 weeks; limb salvage 4/4; no infection/loosening; partial non-union 1 revised.	25% (non-union 1/4)
Fernández et al. [33]	III – Retro cohort (10 PSI/15 Ctrl)	25	Pelvis and sacrum	PSIs vs conventional resection	47 (3-110)	PSI vs Ctrl: R0 80% vs 67%; 5-year OS 60% vs 40%; local recurrence 50% vs 60%; none reached statistical significance (small sample).	PSI: deep infection 40%, superficial 50%; similar deep-infection rate in control
Hu et al. [34]	IV – Retro case series	60	Pelvis	Custom hemipelvic prosthesis + guides	36 ± 15	VAS pain ↓ 5.5→1.7; MSTS ↑ 14.8→23.0; osseointegration 98.5%; local recurrence 6.7%; 5-year OS ≈ 87%.	21.7% overall (poor wound 10%, deep infection 6.7%, dislocation 3.3%)
Lv et al. [35]	IV – Retro case series	12	Sacrum	Two-wing porous sacral implant	38.5 (20-62)	Wide margins 92%; MSTS 21; mean blood loss 3875 mL; 24-month OS 83%; local recurrence 1.	3/12 (25%) total; 1 infection, 2 wound dehiscence
Lv et al. [36]	IV – Retro case series	6	Sacro-iliac joint	Modular sacro-iliac prosthesis	49.8 (18-75)	All negative margins; MSTS 25.3; complete osseointegration 4 months; 0 local recurrences; pulmonary mets 2.	2/6 (33%) delayed wound healing only
Wang et al. [37]	III – Prospective cohort (33 guide/33 Ctrl)	66	Distal femur and proximal tibia	3D surgical guides vs conventional	12	3D group: blood loss 648 ± 138 mL (↓ 6%); resection length 12.6 cm (↓ 0.9 cm); complications 12% vs 33%; MSTS significantly higher at 1, 3, 6, and 12 months; local recurrence 9% vs 15%.	12.1% (3D group) vs 33.3% (control), p = 0.040
Dong et al. [38]	IV – Retro case series	17	Pelvis and extremities	PSIs, anatomic models and custom implants	26.5 (4-69)	R0 63/64 osteotomies (98%); mean OR 10 h; blood loss 1700 ± 1139 mL; MSTS 24 (13-30); 5-year OS ≈ 73%; limb-salvage 100%.	8/17 (47%): 4 wound issues, 3 infections, 1 loosening

Functional Outcomes

Quantitative functional scores were reported in 15 studies encompassing ≈197 patients. Mean MSTS values in individual series ranged from 21 to 28 points (maximum = 30); when weighted by cohort size, the pooled mean was ≈23-24/30, corresponding to ~78-80% of normal limb function. The highest scores (≥27/30) were achieved after ankle, periacetabular, and intercalary femoral reconstructions, while complex pelvic and sacral replacements generally scored in the low- to mid-twenties. Ancillary instruments echoed these findings: talar replacements produced AOFAS scores above 90/100, and the single cervical-spine case doubled its Japanese Orthopaedic Association (JOA) score within 12 months. Unrestricted weight bearing was usually permitted between six and 12 weeks after long-bone or intercalary procedures.

Oncologic Control

Surgical-margin status was documented in 18 studies; negative (R0) margins were obtained in ≥90% of resections in 14 of them. Both comparative cohorts showed higher R0 rates in the 3D arm than in matched conventional controls (80% vs 67% and 98% vs 90%). In aggregate, 23 local recurrences occurred among 296 patients (7.8%). Three pelvic cohorts supplied five-year overall-survival estimates ranging from 60% to 87%, values comparable with contemporary limb-salvage benchmarks. None of the included investigations reported an increased incidence of distant metastasis attributable to 3D technology.

Complications

Fourteen studies provided sufficiently granular adverse-event data. Altogether, 66 of 296 patients experienced at least one complication, yielding an overall incidence of 22%. Wound morbidity predominated: superficial dehiscence or delayed healing occurred in ~9% of all patients, and deep infection in ~8%, with the highest rates (deep up to 40%, superficial up to 50%) after extensive internal hemipelvectomy. Mechanical failures were less common but clinically significant, including implant dislocation (≤27% in porous hemipelvic devices), delayed or non-union after allograft integration (0-33%), and occasional aseptic loosening (≤6%). In the larger level-III cohort, the use of patient-specific guides reduced the total complication rate relative to conventional surgery (12% vs 33%, p = 0.040). Limb salvage was ultimately preserved in every series, and secondary interventions - revision fixation, grafting, or debridement - successfully addressed most mechanical or union-related failures.

Discussion

This systematic review consolidates evidence from 20 investigations involving 296 patients, highlighting the increasingly pivotal role of 3D printing in the surgical management of orthopedic sarcomas. These findings underscore the value of 3D technologies in enhancing surgical accuracy, customizing reconstruction, and potentially reducing morbidity in anatomically complex or high-risk resections. While limitations in study design and heterogeneity exist, several patterns of clinical benefit are apparent.

Achieving negative surgical margins (R0 resection) is a cornerstone of sarcoma surgery and directly correlates with local recurrence and overall survival. The review demonstrates that 3D-assisted surgeries consistently facilitated high rates of R0 resections. In 18 studies that reported margin status, at least 14 achieved negative margins in ≥90% of cases. For instance, Hu et al. [34] reported a 98.5% rate of osseointegration with a local recurrence rate of only 6.7% in their 60-patient hemipelvic cohort. Similarly, Lv et al. [35] and Lv et al. [36] achieved wide margins in 92% and 100% of cases, respectively, using custom sacral and sacroiliac prostheses.

Comparative studies reinforced these observations. In a level-III cohort by Wang et al. [37], the use of 3D-printed surgical guides yielded a significantly higher R0 rate (98%) than conventional resection (90%). Fernández et al. [33] reported similar trends in pelvic and sacral resections, where the R0 margin rate increased from 67% in conventional surgeries to 80% in the PSI group, although statistical significance was limited by small sample size. Overall, only 23 local recurrences were reported across all studies, equating to a recurrence rate of 7.8% - a favorable outcome given the aggressive biology and anatomical challenges of orthopedic sarcomas.

Functional outcomes, measured in 15 studies using instruments like the MSTS score, reflect the success of limb-salvage surgery and quality of life of the patient. Weighted by cohort size, pooled MSTS scores approximated 23-24/30, corresponding to 78-80% of normal limb function. The highest functional scores (≥27/30) were consistently observed in distal limb reconstructions, such as the ankle [32], femur [26], and talus [31], likely due to better mechanical reconstruction and reduced soft tissue disruption.

Conversely, pelvic and sacral reconstructions, despite oncologic success, had slightly lower functional scores, typically in the low- to mid-20s. For example, Bianchi et al. [22] reported a five-year implant survival of 74% and MSTS scores around 24/30, despite the technical difficulty of pelvic surgeries. Xu et al. [26], focusing on porous hemipelvic prostheses, found an average MSTS of 22, with all survivors ambulant at follow-up. Hu et al. [34] also noted a significant improvement from a preoperative MSTS of 14.8 to 23.0 after 3D-assisted resection and reconstruction. In pediatric cases, 3D printing was especially advantageous. Stavrev et al. [17] described a growing porous titanium endoprosthesis that enabled a four-year-old with tibial Ewing sarcoma to regain independent ambulation within three weeks, exemplifying the technology’s adaptability to small or developing anatomies.

Overall, 66 of the 296 patients experienced at least one complication, with an aggregate complication rate of 22%. The majority of adverse events involved soft tissue issues, particularly wound healing complications and infections. Benady et al. [29] reported wound infections in 27% of their hemipelvectomy cases, while Fernández et al. [33] recorded deep infections in 40% and superficial issues in 50% of PSI-assisted resections. These elevated rates likely reflect the extensive exposure and vascularity of pelvic procedures rather than shortcomings of the 3D approach itself.

Mechanical complications, while less frequent, were clinically important. Implant dislocation was reported in porous pelvic reconstructions by Jovičić et al. [20] at a rate of 27%. Graft-related complications such as non-union or delayed healing were noted in hybrid reconstructions by Gasparro et al. [23] (25%) and Benady et al. [29] (17%). However, these complications were typically amenable to secondary interventions like revision fixation or debridement, and none resulted in amputation or complete treatment failure. Importantly, in the comparative study by Wang et al. [37], complication rates were significantly lower in the 3D-assisted group than in the conventional group (12.1% vs 33.3%, p = 0.040), suggesting a potential safety advantage with the use of customized surgical instruments and implants.

Across the included studies, 3D technology was employed in four primary modalities: custom porous titanium implants [18,35], patient-specific hemipelvic or sacral endoprostheses [27,34], resection/cutting guides, and hybrid constructs combining guides with allografts [23,26]. These technologies enabled precise surgical rehearsal, optimal osteotomy design, and improved implant-host integration.

Despite these advantages, intraoperative adjustments were occasionally necessary. Kamat et al. [18] noted that even with meticulous planning, modifications during surgery were needed due to anatomical variability or unanticipated tumor extensions. Moreover, production time and costs remain barriers to broader adoption, particularly in emergency settings or low-resource institutions. Nevertheless, the reproducibility and personalization offered by 3D printing are evident. A study by Benady et al. [29] showcased workflows where resection guides, models, and implants were integrated into a cohesive plan, reducing operating time and radiation exposure while maintaining oncologic rigor.

Limitations and future directions

While the findings are encouraging, the evidence base remains limited by methodological heterogeneity. Most included studies were retrospective case series (level IV), with only two level III comparative cohorts. Follow-up durations varied, and few investigations reported on long-term implant survivorship or cost-effectiveness metrics. Additionally, no study evaluated patient-reported outcome measures or health-economic endpoints, which are essential for value-based care assessments. Looking forward, research should prioritize multicenter prospective cohorts or registry-based evaluations using standardized metrics. The integration of biologically active 3D-printed materials, such as porous scaffolds for osseointegration or drug-eluting prostheses, represents a promising frontier. As regulatory standards and production workflows improve, it is expected that 3D printing will expand its clinical utility not only in sarcoma surgery but across reconstructive orthopedic oncology.

## Conclusions

This systematic review underscores the significant clinical value of 3D printing in the surgical management of orthopedic sarcomas. The technology enables more precise tumor resections and facilitates patient-specific reconstructions, particularly in anatomically challenging regions such as the pelvis and spine. Across diverse clinical contexts, 3D printing has supported limb-salvage procedures, improved functional outcomes, and contributed to favorable oncologic control. Although complications were reported, they were generally manageable, and limb preservation was consistently achieved. Comparative studies suggest that PSIs and custom implants may reduce operative risk and enhance the accuracy of resection compared to conventional techniques. Despite these promising findings, the existing literature remains limited by heterogeneity in study designs, small sample sizes, and the predominance of retrospective evidence. Further research is needed through prospective, multicenter studies that apply standardized outcome measures and assess long-term performance and cost-effectiveness. As clinical experience with 3D printing grows, its integration into orthopedic oncology workflows may continue to refine and personalize surgical care for sarcoma patients.
